# Physiological Constants of the Entomopathogenic Bacterium *Xenorhabdus nematophila* Determined by Microbial Growth Kinetics

**DOI:** 10.1155/2014/834054

**Published:** 2014-04-23

**Authors:** Rinu Kooliyottil, Floyd Inman, Sivanadane Mandjiny, Len Holmes

**Affiliations:** Sartorius Stedim Biotechnology Laboratory, Biotechnology Research and Training Center, University of North Carolina at Pembroke, Pembroke, NC 28372-1510, USA

## Abstract

*Xenorhabdus nematophila*, an entomopathogenic bacterium that symbiotically associates with the entomoparasitic nematode *Steinernema carpocapsae*, was studied to determine its physiological parameters of glucose utilization. *X. nematophila* was cultured in chemically defined media containing various concentrations of glucose under optimal conditions utilizing a two-liter fermentation system. Specific growth rates were obtained from each glucose batch. Specific growth rates and their associated glucose concentrations were used to determine physiological parameters. These parameters include the bacterium's substrate utilization constant (*K*_*s*_) and its maximum specific growth rate (*μ*_max_). The bacteria exhibited a *K*_*s*_ value of 2.02 mg/L suggesting that *X. nematophila* has a high affinity for glucose. The *μ*_max_ of *Xenorhabdus* was determined to be 1.03 h^−1^. Further research is needed to determine if microbial affinities to different substrates have any influence on biological relationships (symbiosis, pathogenicity, parasitism, etc.) between prokaryotes and higher organisms.

## 1. Introduction

*Xenorhabdus nematophila* is a Gram-negative, rod shaped, endosymbiotic bacterium found exclusively within the gut of the entomoparasitic nematode* Steinernema carpocapsae*. Notably,* X. nematophila* is an entomopathogenic member of Enterobacteriaceae and is extremely lethal to its insect host resulting in death within 24–48 hours [1, 2]. In the natural environment, the nematode-bacterial complex is crucial for insect pathogenicity and survival of the symbiotic pair. Due to the pair's interesting biological characteristics, they have become model organisms for studying nematode symbiosis, host-bacterium interactions, and parasitism [3]. These nematode-bacterial complexes are mass produced and commercialized for use as biological insecticides [4, 5].

Inside of* S. carpocapsae*, cells of* X. nematophila* are in a dormant phase and therefore do not proliferate within the nematode gut. To the contrary, once the nematode gains access to the insect hemolymph, regurgitation of its symbiotic bacteria occurs. It is inside of the hemolymph where bacteria proliferation occurs. In order to mass produce entomoparasitic nematodes* in vitro*, it is crucial to understand the physiology of the bacterial symbiont to various substrates that are found within insect hemolymph and potential nematode growth media. In this report, glucose, a primary substrate for growth, proliferation, and function in most organisms, is used to study growth kinetics of* X. nematophila*. To the authors' knowledge there are no reports presently available describing growth and substrate kinetics of* X. nematophila*. In the present study,* X. nematophila* was cultivated in batch mode containing defined media supplemented with various glucose concentrations. Glucose is provided as the sole carbon and energy source within the media. Physiological constants of* X. nematophila* such as the substrate utilization constant (*K*_*s*_) and maximum specific growth rate (*μ*_max⁡_) were determined.

## 2. Materials and Methods

### 2.1. Bacterial Isolation

*X. nematophila* was indirectly isolated on nutrient agar plates streaked with the hemolymph of a nematode-infected larva of* Galleria mellonella*, in similar manner to Inman III and Holmes [6]. Nutrient agar was composed of (g/L) beef extract (3), digested gelatin (5), and agar (15). The identity of the bacterial symbiont was confirmed by several morphological and microscopic techniques [7, 8]. The reference strain* X. nematophila* XQ1 ATCC 39497 was used to compare bacterial characteristics and was obtained from the American Type Culture Collection (Manassas, Virginia, USA).

### 2.2. Kinetics Media

Microbial kinetics of* X. nematophila* was studied in batches of 1.5 liters of defined liquid media containing (mM) NaCl (50), MgSO_4_ (5), KH_2_PO_4_ (50), HEPES (25), NH_4_Cl (30), Na_2_MoO_4_·2H_2_O (0.025), MnCl_2_·4H_2_O (0.025), and FeCl_3_·6H_2_O (0.025) to determine *K*_*s*_ and *μ*_max⁡_. Each batch was supplemented with increasing concentrations of glucose (1.8 mg/L, 9 mg/L, 18 mg/L, and 90 mg/L) to obtain respective growth rates (*μ*). During media preparation, glucose concentrated stocks were prepared and autoclaved separately. The obtained data was further used to determine physiological constants (*K*_*s*_ and *μ*_max⁡_).

### 2.3. Fermentation Parameters and Experiment

Batches were carried out in a two-liter Biostat A plus fermentation system (Sartorius stedim, Germany). Temperature, agitation, pH, and aeration were maintained at 28°C, 200 rpm, 7.30, and 1 vvm, respectively. A vvm is a unit that is equivalent to one volume of gas (liters per minute) added to the one volume of a liquid (L). Optical density was measured with a submersible near-infrared ASD19-N optical density probe (optek-Danulat, Inc., Germany) and measured in concentration units (cu).

Each experimental batch was inoculated at 1.0% concentrations. Overnight cultures were grown within the same chemically defined media with respective glucose concentrations for the upcoming growth experiment. Prior to each inoculation, overnight cultures were washed thrice with phosphate buffered saline, pH 7.2, in order to prevent carryover of any residual glucose from overnight cultures into the growth experiment. Additionally, overnight cultures were harvested in either late deceleration or early stationary phases based upon previous time/growth experiments within the same media (data not shown).

## 3. Results and Discussion

**Figure 1:**
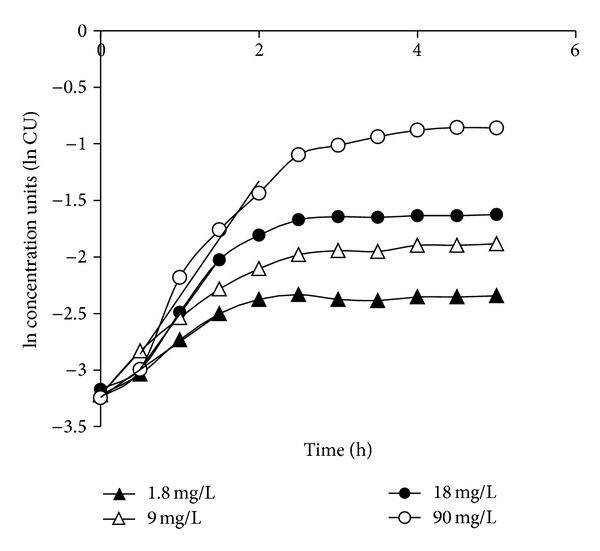
Growth curves of* X. nematophila* in defined medium with varying concentrations of glucose. Each glucose batch resulted in a specific growth rate: 1.8 mg/L (0.49 h^−1^), 9 mg/L (0.78 h^−1^), 18 mg/L (0.97 h^−1^), and 90 mg/L (1.02 h^−1^).

Batch growth curves of* X. nematophila*, for various glucose concentrations (1.8, 9.0, 18.0, and 90.0 mg/L) are depicted in Figure 1. To determine the specific growth rate of each batch, the natural log of concentration units (ln cu) was plotted against time to linearize exponential growth phases [9]. Note that upon taking natural logs of decimals the resultant values are negative; however, taking the absolute value of (ln cu) may be an acceptable practice. Specific growth rates are equivalent to the slope of the linearized exponential growth phase and were determined graphically. Specific growth rates from each glucose batch are reported in order of increasing glucose concentrations: 1.8 mg/L (0.49 h^−1^), 9.0 mg/L (0.78 h^−1^), 18 mg/L (0.97 h^−1^), and 90 m g/L (1.02 h^−1^).

**Figure 2:**
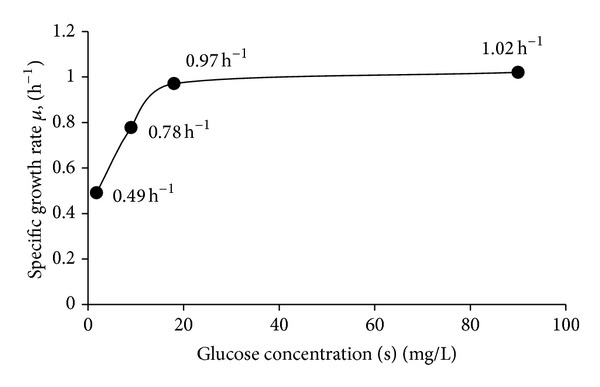
Monod plot of specific growth rates of* X. nematophila* as function of varying glucose concentrations. Notice how the plot approaches to an asymptotic value. The value of this upper asymptote, as described by Monod, is equivalent to the maximum specific growth rate (*μ*_max⁡_) of the organism when substrate concentrations are not limiting [10].

Monod stated that the specific growth rate (*μ*) of a bacterial culture is dependent upon the nutrient concentration [*s*] and can be modeled as seen in (1) below [10]. The model plots various substrate concentrations and their resultant specific growth rates as a consequence; a hyperbolic function is observed. The slope of the function is equivalent to the maximum specific growth rate (*μ*_max⁡_) divided by the sum of the substrate utilization constant (*K*_*s*_) and the substrate concentration used [*s*]. By observing the Monod plot of the study's data, µ did not increase significantly (~0.05 h^−1^) when glucose was increased from 18 mg/L to 90 mg/L (Figure 2):

(1) $$\begin{array}{c} \mu =\frac{\mu_{\max}}{K_{s}+\left[s\right]}\cdot \left[s\right]. \end{array}$$

Equation (1) shows the Monod equation relating the specific growth rate (*μ*) to substrate concentration ([*s*]). The terms *μ*_max⁡_ (maximum specific growth rate) and *K*_*s*_ (substrate utilization constants) are physiological constants of the organism and are determined mathematically.

The growth data shown in Figure 2 was utilized to calculate values of glucose *K*_*s*_ and *μ*_max⁡_ constants for* X. nematophila*. To mathematically determine the constants, the Monod equation was first linearized. Since the Monod equation is similar to the Michaelis-Menton equation used in enzyme kinetics, the same linearization models of the Michaelis-Menton equation can be used. From the three linearization models (Lineweaver-Burk, Eadie-Hofstee, and Hanes-Wolfe), the Eadie-Hofstee linearization (2) was used for various reasons [11]:

(2) $$\begin{array}{c} \mu =-K_{s}\left(\frac{\mu }{\left[s\right]}\right)+\mu_{\max}. \end{array}$$

Equation (2) shows the Eadie-Hofstee linearization of the Monod equation. The resultant linearization of the Monod equation simply depicts the slop as the negative *K*_*s*_ value with a *y*-intercept of *μ*_max⁡_. This is true when the specific growth (*μ*) is plotted as a function of the same specific growth rate divided by its corresponding substrate concentration ([*s*]).

**Figure 3:**
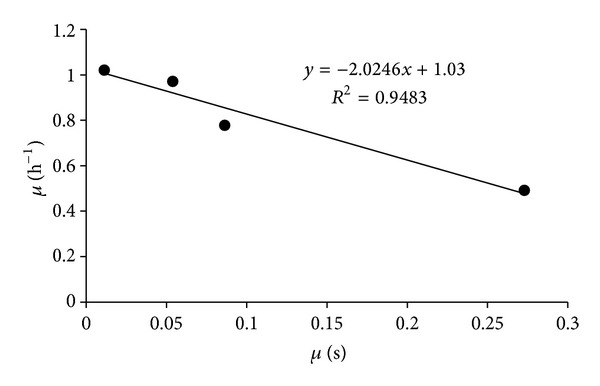
Eadie-Hofstee linearization plot to calculate the physiological constants *K*_*s*_ and *μ*_max⁡_. By plotting *μ* as a function of *μ*/[*s*], the resulting slope is equivalent to the negative value of *K*_*s*_; therefore, the glucose *K*_*s*_ value for* X. nematophila* is reported to be 2.02 mg/L. According to (2), *μ*_max⁡_ is defined as the *y*-intercept; therefore, *μ*_max⁡_ for* X. nematophila* was calculated to be 1.03 h^−1^.

The glucose *K*_*s*_ was obtained from the slope of the Eadie-Hofstee diagram by plotting µ as a function of *μ*/[*s*]. According to the Eadie-Hofstee plot, the glucose *K*_*s*_ and *μ*_max⁡_ values were mathematically determined to be 2.02 mg/L and 1.03 h^−1^, respectively (Figure 3). At this time, there are no other publications that report physiological constants (*K*_*s*_ and *μ*_max⁡_) for* X. nematophila* and only one publication exists for* Photorhabdus luminescens*, another entomopathogenic bacterium associated with the entomoparasitic nematode* Heterorhabditis bacteriophora* [9]. Bowen et al. reported the glucose *K*_*s*_ for* P. luminescens* (1.95 mg/L) and *μ*_max⁡_ (0.96 h^−1^) [9].

## 4. Conclusion

The present study is the initiation of understanding the physiological nature of the entomopathogenic bacterium* X. nematophila* in regard to glucose utilization within the insect hemolymph. Similar research involving different substrates may provide clues to understand how* X. nematophila* and other bacterial symbionts of entomoparasitic nematodes utilize the nutrients found within host insect hemolymph. Some of the nutrients found within insect hemolymph are obviously utilized for bacterial growth and proliferation that may also be involved with insect pathogenicity [9]. Monod describes *K*_*s*_ as a measure of microbial affinity towards the substrate [10]. Monod adds that the lower the *K*_*s*_ value is the higher the microbial affinity is towards that particular substrate. Additionally, in regard to *μ*_max⁡_, Monod states that once a culture's specific growth rate reaches that of the maximum specific growth rate, the rate will no longer increase regardless if substrate concentration is increased [10]. Monod also offers conditions that result in a growth rate that is equivalent to *μ*_max⁡_; then, substrate concentrations are not limited and can support maximum population density.

Future research is required to understand how affinities to different substrates can be associated with different relationships (symbiotic, pathogenic, parasitic, etc.) between prokaryotes and higher organisms. In this study, the glucose *K*_*s*_ value was found to be very low at 2.02 mg/L, which suggests that* X. nematophila* has a high glucose affinity. In comparison to* P. luminescens*, the reported glucose *K*_*s*_ was 1.95 mg/L, suggesting that the *K*_*s*_ determined for* X. nematophila* is within range [9]. Bowen et al. speculate that this high affinity towards glucose may be useful as a biological indicator of pathogenicity that may correlate to how quickly these entomopathogenic bacteria can proliferate in nonlimiting conditions within the insect host [9].
